# The Beat

**Published:** 2006-08

## Tax Schemes for Environmental Payoff

**Figure:**
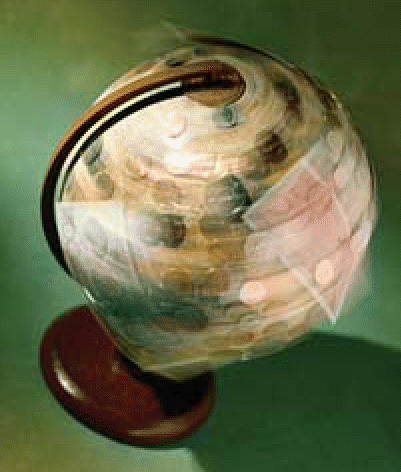


A new policy brief from the World Resources Institute and the Brookings Institution examines how different fiscal strategies can both raise money and benefit the environment. The brief discusses state-level initiatives that tax septic systems and gasoline consumption as well as the federal law signed in 1989 that taxes certain ozone-depleting chemicals. This law brought about the 38% reduction in use of those chemicals in the year 1990 and raised almost $3 billion in its first five years. The brief also points out tax schemes that have had unintended adverse environmental effects. The authors propose water pollution, nitrogen fertilizer, and carbon as viable options for taxation. The brief is available online at http://pdf.wri.org/greening_the_tax_code.pdf.

## WTO Kills European GMO Moratorium

In May 2006, the World Trade Organization ruled that the European Union moratorium on genetically modified (GM) foods was illegal. The case was brought by the United States, Canada, and Argentina, the world’s biggest producers of GM foods. The ruling also came down against six individual European member states that had their own bans on certain GM products, stating they had provided no scientific evidence to justify their moves. The case did not address the safety of GM foods or whether they can be compared to conventional products. The ruling can be appealed by both parties.

## Ironic Breeze

**Figure:**
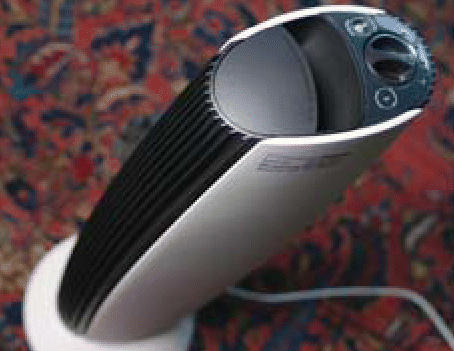


Researchers at the University of California, Irvine, confirm in the May 2006 issue of the *Journal of the Air & Waste Management Association* that indoor air purifiers used in small, poorly ventilated areas can add to indoor ozone levels, creating concentrations that exceed regulatory standards. In the study, ozone levels reached levels higher than 350 ppb, which would trigger a Stage 2 smog alert if it occurred outdoors. Ozone can cause lung damage and aggravate chronic lung diseases such as asthma. No agency has the authority to govern the amount of ozone that air purifiers can produce. However, the U.S. EPA and the California Air Resources Board have issued advisories discouraging the use of these machines.

## Sunscreen Ads Miss Men

**Figure:**
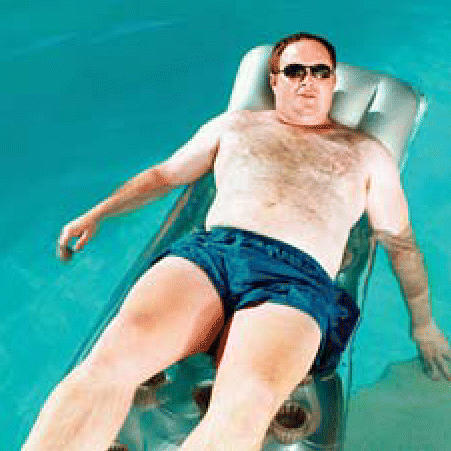


A Boston University review of 24 popular magazines found that publications aimed at groups at high risk for skin cancer rarely contain advertising for sun protection products. Middle-aged and older men are both the least likely to use sunscreen and the most likely to die from melanoma, the deadliest form of skin cancer. But of almost 800 sun-care product ads that appeared in six years’ worth of the 24 magazines, three-quarters were found in women’s magazines. The researchers noted that women’s magazines ran an average of four sun-care product ads per issue, while parenting and family magazines carried less than one per issue, and outdoor recreation magazines aimed at men ran ads just once every six issues.

## Wal-Mart Aims for Organic

The summer of 2006 will see the food shelves of the world’s largest retail chain, Wal-Mart, getting an organic boost. The company will begin selling a wide range of organic foods at relatively affordable prices—possibly just 10% higher than conventional food. Wal-Mart, already the biggest seller of organic milk, is now pressing its suppliers for organic versions of well-known brand-name products. Critics worry that the move will force more industrialization of organic farming in ways that may not be true to traditional organic principles—for example, by forgoing the field rotation used by small farms. Further, because supply for organic goods already lags behind demand, Wal-Mart may have to turn to suppliers overseas, which will cause more transportation-related pollution.

## Random Acts of Sustainability

**Figure:**
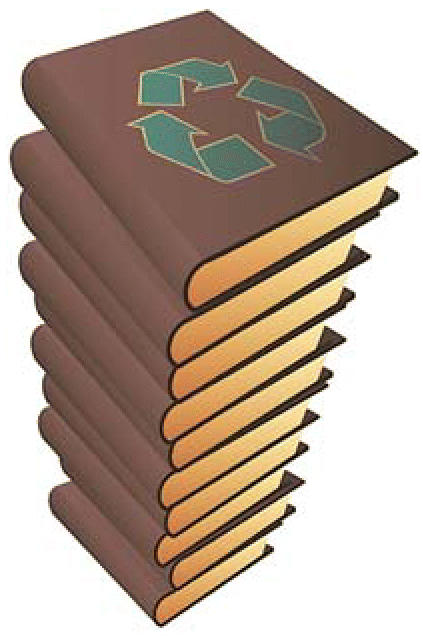


Random House, a publisher with 13% of the U.S. adult book trade, announced in May 2006 that it plans to raise the amount of recycled paper it uses to print books from 3% to 30% by the year 2010. Random House is the first major U.S. publisher to commit to such a change. By 2008, the company also aims to use at least 10% recycled materials for glossy items such as art and cookbooks. More than 500,000 trees could be saved yearly thanks to the switch. Luckily for book buyers, the cost for switching to recycled paper should be in the range of cents, not dollars.

